# Acquisition of a Leucine Zipper Motif as a Mechanism of Antimorphy for an Allele of the *Drosophila Hox* Gene *Sex Combs Reduced*

**DOI:** 10.1534/g3.114.010769

**Published:** 2014-03-12

**Authors:** Lovesha Sivanantharajah, Anthony Percival-Smith

**Affiliations:** Department of Biology, The University of Western Ontario, London, Ontario 5A6 5B7, Canada

**Keywords:** *Homeotic selector* genes, body patterning, *Sex combs reduced*, evolutionarily conserved protein motifs, leucine zipper, interaction interface, antimorph, dominant negative

## Abstract

In 1932, Müller first used the term "antimorphic" to describe mutant alleles that have an effect that is antagonistic to that of the wild-type allele from which they were derived. In a previous characterization of mutant alleles of the *Drosophila melanogaster Hox* gene, *Sex combs reduced* (*Scr*), we identified the missense, antimorphic allele *Scr*^14^, which is a Ser_10_-to-Leu change in the N-terminally located, bilateran-specific octapeptide motif. Here we propose that the cause of *Scr*^14^ antimorphy is the acquisition of a leucine zipper oligomerization motif spanning the octapeptide motif and adjacently located protostome-specific LASCY motif. Analysis of the primary and predicted secondary structures of the SCR N-terminus suggests that while the SCR^+^ encodes a short, α-helical region containing one putative heptad repeat, the same region in SCR^14^ encodes a longer, α-helical region containing two putative heptad repeats. In addition, *in vitro* cross-linking assays demonstrated strong oligomerization of SCR^14^ but not SCR^+^. For *in vivo* sex comb formation, we observed reciprocal inhibition of endogenous SCR^+^ and SCR^14^ activity by ectopic expression of truncated SCR^14^ and SCR^+^ peptides, respectively. The acquisition of an oligomerization domain in SCR^14^ presents a novel mechanism of antimorphy relative to the dominant negative mechanism, which maintains oligomerization between the wild-type and mutant protein subunits.

Body patterning in bilaterans requires the *Homeotic selector* genes (*Hox*) (Lewis 1978; McGinnis and Krumlauf 1992; Carroll 1995). In *Drosophila melanogaster*, *Hox* genes are expressed in spatially restricted domains along the anterior–posterior axis of the embryo and function as sequence-specific, DNA-binding transcription factors to establish the unique identity of each body segment. One focus in the study of *Hox* genes is to understand how the evolution of *Hox* regulation of development has contributed to the morphological variation observed in Bilateria (Carroll *et al.* 2005). Many previous studies have examined the contribution of *cis*-regulatory motifs to changes in *Hox* function; however, our understanding of HOX protein structure and functional contribution of highly conserved motifs and domains to HOX activity is limited. Functionally important regions are often synonymous with highly conserved proteins domains, which are the products of purifying selection; therefore, these regions offer a starting point for the dissection of protein function. In the study of Drosophila HOX protein structure, the greatest emphasis has been placed on identifying the developmental roles of two HOX protein motifs found in all Bilaterans: the homeodomain (HD) and the YPWM motif (Zhao *et al.* 1996; Curtis *et al.* 2001; Galant *et al.* 2002; Hittinger *et al.* 2005). Surprisingly, although both of these regions are important for HOX function, the only essential domain is the DNA-binding HD (Berry and Gehring 2000; Tayyab *et al.* 2004; Joshi *et al.* 2007). An explanation for this finding is the observation of differential pleiotropy: the additive, context-dependent function of HOX protein motifs and domains (Hittinger *et al.* 2005; Prince *et al.* 2008; Sivanantharajah and Percival-Smith 2009; Merabet *et al.* 2011; Percival-Smith *et al.* 2013).

Here, we are interested in identifying the regions that have been functionally conserved in the HOX protein, Sex combs reduced (SCR). SCR has 10 protein motifs and domains conserved at different taxonomical levels: bilateran-specific (octapeptide, YPWM, HD, and KMAS); protostome-specific or arthropod-specific (LASCY, SCKY, PQDL, and NANGE); and insect-specific (DTYQL and C-terminal domain) (Percival-Smith *et al.* 2013). During development, SCR establishes the identity of the labial and prothoracic segments. In the labial segment, SCR function is required for development of the proboscis, which is the adult feeding tube, and the larval salivary glands; however, in the prothoracic segment, SCR is required for establishing the identity of the prothoracic legs, which are characterized by the presence of sex combs on the fifth tarsal segment (Lewis *et al.* 1980; Struhl 1982; Panzer *et al.* 1992; Percival-Smith *et al.* 1997). The previously identified missense, antimorphic allele, *Scr*^14^, is a Ser_10_ to Leu substitution of the most conserved residue in the SSYF submotif of the octapeptide motif (Tour *et al.* 2005; Sivanantharajah and Percival-Smith 2009). The analysis of the interaction between *Scr^14^* and *Scr*^+^ and the interaction between *Scr^14^* and null alleles led to the classification of this allele as an antimorph, which is a class of mutant alleles that are antagonistic to the wild-type allele (Müller 1932; Sivanantharajah and Percival-Smith 2009). The subsequent interpretation of this genetic evidence led to the proposal that the octapeptide motif of SCR may mediate dimerization between SCR N-termini (Sivanantharajah and Percival-Smith 2009).

A common interaction motif found in the bZIP and the bHLH-ZIP classes of transcription factors is the leucine zipper motif (Vinson *et al.* 1989; Murre *et al.* 1989). These proteins, when bound to DNA, are long α-helices with N-terminal DNA binding domains and C-terminal dimerization domains that form a leucine zipper coiled coil (Landschultz *et al.* 1988; Vinson *et al.* 1989; Ellenberger *et al.* 1992). The leucine zipper regions in these transcription factors typically comprise four or more heptad repeats; however, there is growing evidence that shorter and moderately stable leucine zipper motifs, consisting of only two heptad repeats, can function independently as oligomerization domains (Burkhard *et al.* 2000; Perera *et al.* 2001; Nikolaev and Pervushin 2009). The leucine zipper heptad contains seven amino acids with a standard notation of (*a b c d e f g*)_n_ (Krylov *et al.* 1994). Coiled coil interactions promote oligomerization between the leucine zipper motifs of one protein and its partner, and the partner’s heptad repeats are designated (*a′ b′ c′ d′ e′ f′ g′*)_n_. The structure and biochemical properties of the coiled coil structure formed by the interaction of two leucine zipper motifs are dependent on the intermolecular interactions that occur between the hydrophobic, stability-conferring residues in positions *a-a′* and *d-d′*, and the interaction between charged, specificity-conferring residues in positions *g-e′* (Krylov *et al.* 1994). Because the amino acid at position *g* of one heptad interacts with the amino acid occupying position *e′* in the adjacent heptad of the dimerization partner, the designation (*g a b c d e f*)_n_ accounts for all relevant intermolecular interactions. Here we report that the antimorphic–hypomorphic *Scr*^14^ allele may be the result of the acquisition of a short, functional leucine zipper motif spanning two evolutionarily conserved motifs in the SCR N-terminus: the bilateran-specific octapeptide motif and the adjacently located, protostome-specific LASCY motif.

## Materials and Methods

### *Ab initio* method for prediction of secondary structure of the SCR^+^ and SCR^14^ N-termini

The Protein Data Bank (PDB) was searched with two queries: the wild-type sequence of the SCR N-terminus (FAMSSYQFVNSLACYPQMN) and the sequence of SCR^14^ (FAMS**L**YQFVNSLACYPQMN). All peptide sequence matches were assessed using the following criteria: peptide sequences of six or more amino acids in length were used, except if a sequence of five amino acids had perfect identity and all matches with insertions or deletions were ignored; to improve the accuracy of identifying homologous sequences without perfect identity, the BLOSUM62 substitution matrix (Henikoff and Henikoff 1992) was applied. Matches with amino acid substitution scores that equaled zero or above were used. If two substitutions were present, then homologous peptide sequences were used only if the substitutions had positive scores; matches with the same peptide sequences were used if the proteins were from different protein families. Once a match was identified, Cn3D (NCBI) was used to identify the secondary (2°) structure encoded by a protein sequence (*i.e.*, α-helix, β-strand, coil, turn) within a protein with solved 3D structure.

### Expression and affinity purification of SCR peptides from *Escherichia coli*

For biochemical analyses, *Escherichia coli* expression constructs were made for the purification of triple-tagged (TT) (composed of 6xHis, Strep, and Flag tags) (Tiefenbach *et al.* 2010), 29-amino-acid-long SCR peptides, SCR^+^ (SCR^SL^), SCR^14^ (SCR^LL^), SCR^LS^, and SCR^SS^, by inserting the relevant coding sequence into the multiple cloning site of pET-3a (Studier and Moffatt 1986; Studier *et al.* 1990). All constructs were transformed into BL21(DE3) cells (Studier and Moffatt 1986) and protein expression was induced using the Novagen toxic cells protocol. Tagged SCR peptides were purified from *E. coli* using Strep-Tactin Sepharose (IBA) according to the manufacturer’s protocol.

### Protein cross-linking and Western analysis

Purified proteins were incubated without and with (1%) the protein cross-linker, formaldehyde, for 10 min at room temperature. Samples were heated to 65° in SDS buffer for 8 min and run on 12%–15% SDS PAGE. Immobilon-P transfer membrane (Millipore) was used for Western blotting. The anti-FLAG mouse monoclonal antibody (Sigma) was diluted 1:40,000. Antibody–antigen complexes were detected with anti-mouse HRP-conjugated secondary antibody (Sigma) and the SuperSignal West Femto Chemiluminescent kit (Pierce). Images were collected on a Fluorchem 8900 gel documentation system (Alpha Innotech).

### Transgenic flies

Transgenic lines were created to express full-length and truncated SCR proteins fused with a C-terminal triple-tag using the GAL4-UAS system (Brand and Perrimon 1993). All constructs were made and independently incorporated into the Drosophila genome using *P*-element–mediated transformation (Rubin and Spradling 1982). Full-length (SCR_FL_TT) proteins with wild-type (SCR^+^) and the SCR^14^ sequence and were expressed in all three leg discs using the *rotund* (*rn*)-*Gal4* (*P{GawB}*) driver. Truncated SCR peptides encoded the first 121 amino acids of the SCR N-terminus with wild-type sequence, specific deletions, singular or in combination, of the first eight amino acids of SCR (8aa), the octapeptide (octa), and the LASCY motif (LASCY), and point mutations of Ser_10_ to Leu, as in SCR^14^, and Ser_10_ to Ala. Fly lines of the genotype, *y w*; *P{*UAS-*Scr^x^*_121_TT, *w^+^}*; *pb*^34^/TM6B, were established and crossed to either *P{Armidillo* (*Arm*)-*Gal4*, *w^+^}*; *Scr*^+^/TM6B or *P{Arm-Gal4*, *w^+^}*; *Scr*^14^/TM6B driver lines to produce progeny in which UAS constructs were constitutively expressed in *Scr*^+^ or *Scr*^14^ hemizygotes; these flies have one endogenous copy of *Scr* because *pb*^34^ is a deficiency encompassing the *Scr* locus. Flies of genotype *P*{*Arm-Gal4*, *w^+^*}; *Scr*^+^/*pb*^34^ and *P{Arm-Gal4*, *w^+^}*; *Scr*^14^/*pb*^34^ were used as controls for GAL4 expression. Three phenotypes were quantified: the number of sex comb bristles on the T1 leg; the number of pseudotrachea that developed on the proboscis; and the number of nuclei in the salivary glands. A description of the genetic markers and balancer chromosomes used can be found elsewhere (Lindsley and Zimm 1992).

### Expression of UAS constructs *in vivo*

Protein was extracted from third instar larvae of the genotype *y w*; *P{UAS-Scr^x^_121_TT*, *w^+^*}/ *P*{*Arm-Gal4*, *w^+^*}; *Scr*^+^/*pb*^34^. The FLAG epitope tag was detected with the anti-FLAG antibody. Tubulin was used as a loading control and was detected using anti-β-tubulin E7 mouse monoclonal antibody (Klymkowsky *et al.* 1987). SCR peptide and tubulin expression levels were quantified from three independent Western blots using the AlphaEase Fluorchem software (v.4.0.1).

### Statistical analyses

All data were analyzed in SPSS v.20.0 (SPSS Inc. 2011). Comparisons of phenotypic data collected in 2013 and 2009 (Sivanantharajah and Percival-Smith 2009) for the mean number of sex comb bristles, pseudotracheal rows, and nuclei in the salivary gland were peformed using Student *t* tests. Analyses of multiple means were performed using ANOVA and Kruskal-Wallace tests and, if significant differences were detected, then multiple pair-wise comparisons were performed using Tukey tests and Dunnett T3 tests, respectively. In characterizing *Scr*^14^ antimorphy *in vivo* using full-length proteins, data for number of sex comb bristles on the T1 leg were log_10_-transformed to meet the requirements of homoscedasticity and normality and then analyzed using a one-way ANOVA. The data for number of sex comb bristles on the T2 and T3 legs were analyzed using Student *t* tests. In characterizing *Scr*^14^ antimorphy *in vivo* using truncated peptides, all salivary gland data were analyzed using one-way ANOVA. All data for number of sex comb bristles in *Scr*^+^ hemizygotes and pseudotrachea in *Scr*^14^ hemizygotes were analyzed using one-way ANOVA; the latter data set was log_10_-transformed first. All data for number of sex comb bristles in *Scr*^14^ hemizygotes and pseudotrachea in *Scr*^+^ hemizygotes were analyzed using Kruskal-Wallace tests. In mapping the region of SCR required for antimorphy, sex comb bristle and salivary gland data were analyzed using one-way ANOVA. UAS-SCR peptide expression data were square root–transformed and analyzed using a one-way ANOVA.

## Results

### *Scr*^14^ is an antimorphic–hypomorphic allele

**Figure 1:**
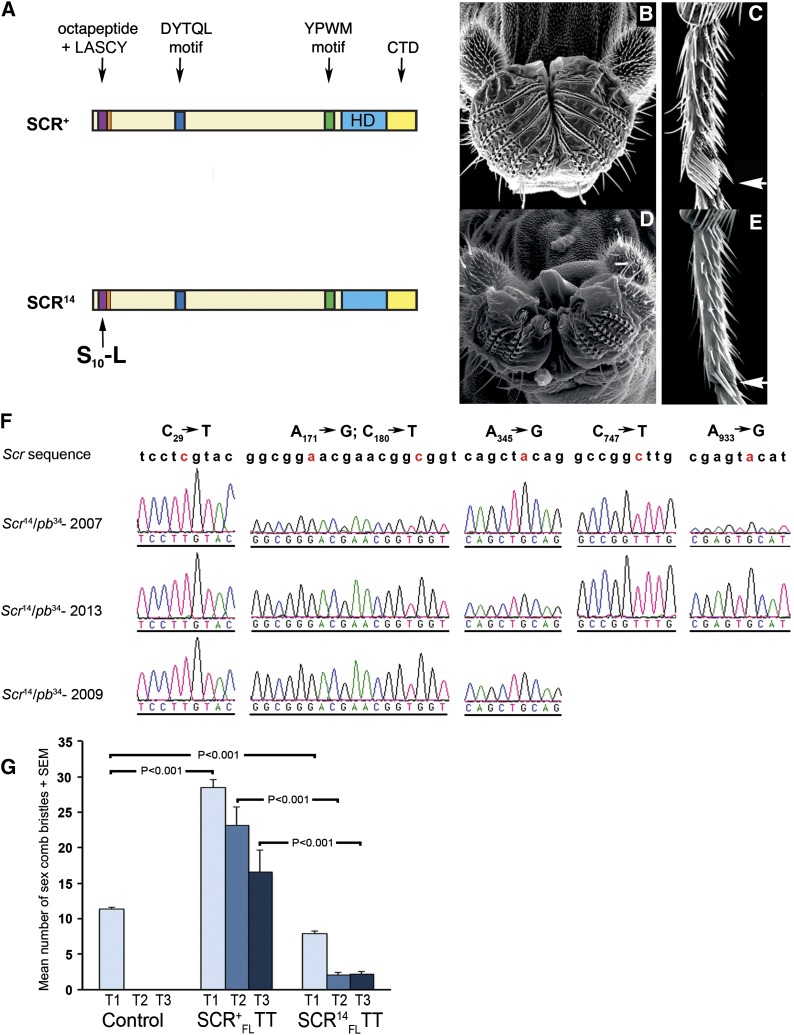
Proboscis and sex comb phenotypes of the antimorphic–hypomorphic allele, *Scr*^14^. *Scr*^14^ is a missense allele in the highly conserved, octapeptide motif. (A) Representations of the SCR proteins encoded by *Scr*^+^ and *Scr*^14^ alleles are shown. Scanning electron micrographs of the adult labial palps and of the fifth tarsal segment of the adult prothoracic leg of flies hemizygous for *Scr*^+^ (B, C) and *Scr*^14^ (D, E). When compared to the *Scr*^+^ hemizygotes, *Scr*^14^ flies show a decreased number of rows of pseudotrachea and sex comb bristles, indicating that *Scr*^14^ is a hypomorphic allele. The arrows indicate the position of the sex comb. Images of *Scr*^+^ flies were taken from Sivanantharajah and Percival-Smith (2009). (F) Electropherogram sections illustrating the missense (C_29_→T) and five silent mutations (A_171_→G, C_180_→T, A_345_→G, C_747_→T, and A_933_→G) initially identified in exon 2 of *Scr*^14^. (G) Expression of full-length SCR^+^_FL_TT and SCR^14^_FL_TT proteins in the three legs (T1–T3) demonstrates that SCR^14^ is both antimorphic and hypomorphic. The numbers of sex comb bristles that form on the three legs are shown for expression from the *rn-Gal4* driver of GAL4 alone (control), SCR^+^_FL_TT, and SCR^14^_FL_TT. *P* values listed above black lines indicate the results of specific pair-wise comparisons.

*Scr*^14^ was initially classified as an antimorphic allele based on the observation that *Scr*^14^/*Scr*^+^ had the same phenotype as *Scr*^14^/*pb*^34^ or *Scr*^+^/*pb*^34^. In addition, the observation that *Scr*^+^/*pb*^34^ and *Scr*^14^/*pb*^34^ had the same phenotype was the major observation for the proposal that SCR^+^ and SCR^14^ formed an inactive locked complex *in vivo* (Sivanantharajah and Percival-Smith 2009). Reexamination of the *Scr*^14^ allele on *Scr*-dependent phenotypes in this study has revealed a change in allelic behavior. When the phenotypes observed now were compared to the phenotypes observed in 2009, the mean number of sex comb bristles in *Scr*^14^ hemizygotes decreased from 7 to 2.5 (t_(64)_=14.6; *P* < 0.001), the mean number of pseudotrachea decreased from 5.4 to 3.0 [t_(12)_= 6.9; *P* < 0.001], and the mean number of nuclei in the salivary glands decreased from 114.8 to 100.5 [t_(30)_=3.2; *P* = 0.0030] (Table 1). We sequenced the *Scr*^14^ allele over the deficiency *pb*^34^ at three different times independently: in 2007 for initial sequencing analysis, in 2009 to confirm the genotype of *Scr*^14^/*pb*^34^ flies that had a wild-type phenotype, and in 2013 (Figure 1). The missense change, causing Ser_10_ to Leu in the octapeptide motif, and all polymorphisms in exon 2 that were reported in 2007 are still present in the 2013 sequence. Despite the observed differences in *Scr*^14^ phenotype, the *Scr*^14^/*Scr*^+^ hemizygote still had the lowest number of sex comb bristles of all other viable *Scr* hypomorphic alleles with *Scr*^+^ (Table 1) (Sivanantharajah and Percival-Smith 2009). If no interaction occurred between these two alleles, then one would expect the sum total of their individual activity to produce 8.7 sex comb bristles and not 7.0 (Table 1). This indicates that *Scr*^14^ inhibits *Scr*^+^ activity and is therefore antimorphic. Second, we observed an inhibitory interaction between *Scr*^14^ and *Scr*^13A^. *Scr*^13A^ is a null allele encoding a truncated protein product of 260 amino acids, where the HD and CTD have been deleted but the N-terminal region, encoding the octapeptide and LASCY motifs, is still present. If no interaction occurred between *Scr*^13A^ and *Scr*^14^, then one would expect the sum total of their individual activity to be the same as *Scr*^14^ alone (*Scr*^14^/*pb*^34^) because *Scr*^13A^ is a null allele that should be functionally equivalent to *pb*^34^; however, *Scr*^14^/*Scr*^13A^ heterozygotes showed a significant decrease in sex comb bristles when compared to *Scr*^14^/*pb*^34^ hemizygotes of 2.5 to 1.9 [t_(97)_=3.6; *P* = 0.001] (Table 1 in italics). Finally, overexpression of SCR^+^_FL_TT and SCR^14^_FL_TT proteins using the *rn-Gal4* driver and UAS fusion genes in the T1 segment had a significant effect [F_(2)_=278; *P* < 0.001] (Figure 1G). In control flies, when GAL4 protein is expressed alone in the leg discs, a mean number of 11.3 ± 0.3 sex comb bristles develop on the first thoracic leg (T1). When SCR^+^_FL_TT is expressed, there is a significant increase in the mean number of sex comb bristles on T1 from 11.3 ± 0.3 to 28.5 ± 1.1 (*P* < 0.001). In contrast, expression of SCR^14^_FL_TT results in a significant decrease in the number of sex comb bristles on the T1 leg from 11.3 ± 0.3 to 7.9 ± 0.4 (*P* < 0.001). This indicates that SCR^14^_FL_TT expression in T1 inhibits endogenous SCR activity, supporting the classification of *Scr*^14^ as an antimorphic allele.

**Table 1:** Comparison of *Scr* phenotypes observed in this study and in a previous study (±SEM)

Genotype	Mean Sex Comb Bristles, No.		Mean Pseudotrachea, No.		Mean Cells in Salivary Gland, No.	
	2013	2009^1^	2013	2009	2013	2009
*Scr^+^/pb^34^*	6.2 ± 0.1	6.3 ± 0.2	5.3 ± 0.1	5.5 ± 0.1*	108.7 ± 3.1	117.7 ± 3.9
*Scr^14^/pb^34^*	2.5 ± 0.1	7.0 ± 0.3*	3.0 ± 0.1	5.4 ± 0.3*	100.5 ± 3.2	114.8 ± 3.2*
*Scr^14^/Scr^+^*	7.0 ± 0.1	6.9 ± 0.2	6.0 ± 0.1	5.5 ± 0.1*	110.1 ± 3.3	112.8 ± 3.0
*Scr^14^/Scr^13A^*	1.9 ± 0.1	2.6 ± 0.1*	4.8 ± 0.2	5.2 ± 0.1	102.5 ± 4.5	123.6 ± 2.7*
*Scr^14^/Scr^6^*	7.1 ± 0.2	6.3 ± 0.2*	6.0 ± 0.0	5.9 ± 0.1*	108.4 ± 3.1	126.0 ± 5.9*

1 Values taken from Sivanantharajah and Percival-Smith (2009).

* Significant differences for a particular phenotype examined in 2009 from values collected in 2013 (*P* < 0.05).

Two observations show that *Scr*^14^ is also hypomorphic. When compared to the *Scr*^+^ hemizygotes, *Scr*^14^ flies show a decreased number of rows of pseudotrachea and sex comb bristles (Figure 1, A–D). Second, relative to full-length SCR^+^_FL_TT, expression of full-length SCR^14^_FL_TT in the T2 and T3 segments with the *rn-Gal4* driver had a decreased capacity to induce ectopic sex comb bristles (Figure 1G). In control flies, when GAL4 protein is expressed alone, no sex comb bristles develop on the T2 and T3 legs. When SCR^+^_FL_TT is expressed, there is an increase to 23.1 ± 2.7 sex comb bristles on T2 and to 16.5 ± 3.1 bristles on T3. Compared to SCR^+^_FL_TT, expression of SCR^14^_FL_TT results in a significant decrease in ectopic sex comb bristles to 2.1 ± 0.3 on T2 [t_(13)_=7.9; *P* < 0.001] and to 2.23 ± 0.4 on T3 [t_(16)_=4.6; *P* < 0.001]. Relative to SCR^+^, SCR^14^ has reduced function, which is the definition of hypomorphy. All evidence together suggests that *Scr*^14^ in 2013 is better classified as an antimorphic–hypomorphic allele. The locked complex model previously proposed is no longer applicable because it was dependent on SCR^+^ and SCR^14^ having wild-type activity when alone but forming inactive heterodimers when together (Sivanantharajah and Percival-Smith 2009).

### The octapeptide and LASCY motifs may comprise a putative leucine zipper motif in SCR^14^

**Figure 2:**
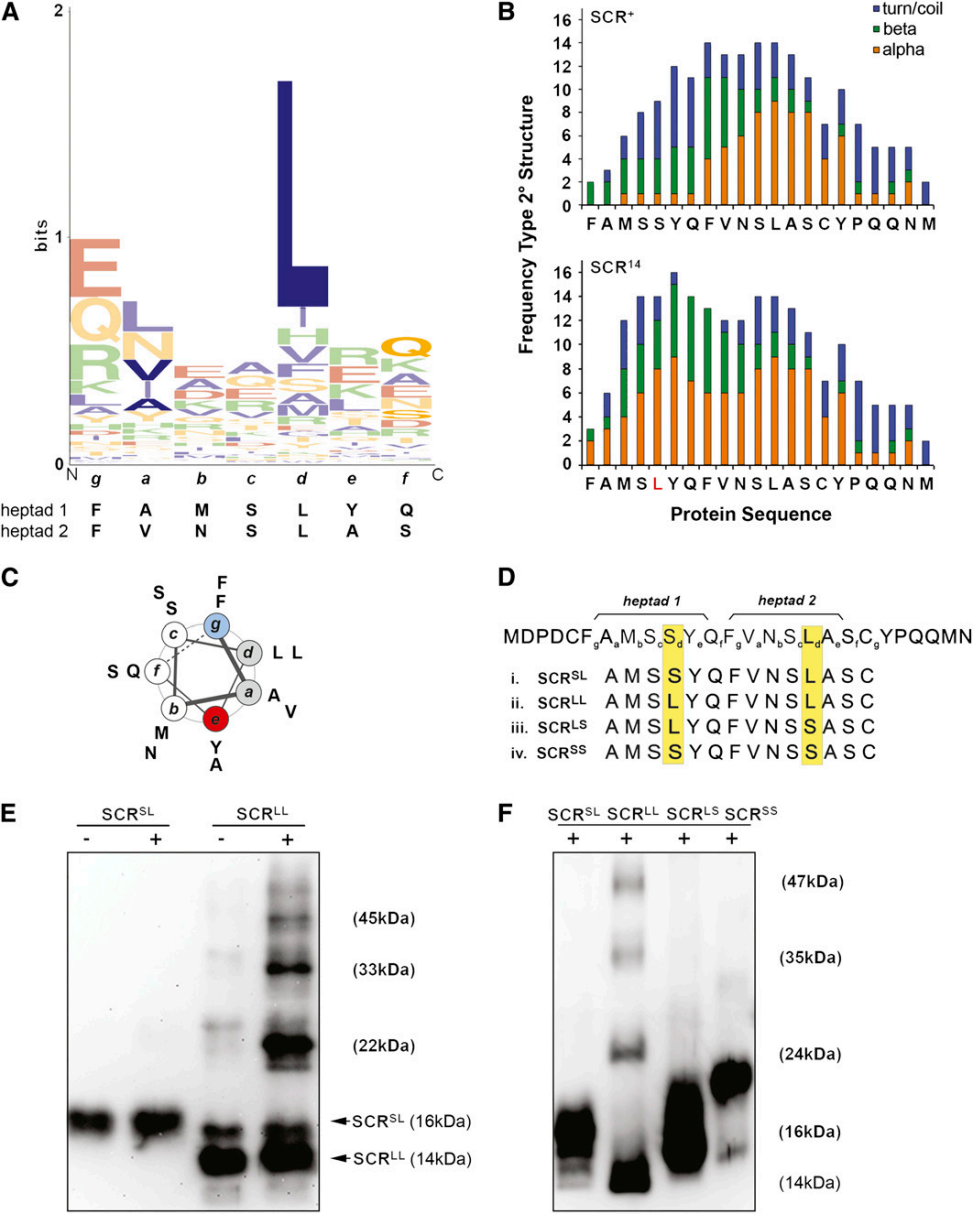
The N-terminus of SCR^14^ satisfies the criteria for a leucine zipper motif. (A) General consensus of a heptad repeat of a leucine zipper motif in Drosophila. The sequence logo was made from the sequences of 130 heptad sequences from 27 Drosophila bZIP proteins that were identified and aligned in the study by Fassler *et al.* (2002). Amino acids have been color-coded according to their biochemical properties. Basic residues are green (K, R, H), acidic residues are red (D, E), hydrophobic residues are blue (G, A, V, L, I, M, P, F, W), and hydrophilic residues are orange (S, T, N, Q, Y, C). The *x*-axis displays the general consensus for a heptad repeat (*g a b c d e f*)_n_ of a Drosophila bZIP protein, which is ELEALRQ. The amino acids of the heptad are displayed from N-terminal (N) to C-terminal (C). The *y*-axis is a measure of uncertainty (bits). The height of a stack indicates the degree of sequence conservation at that position, and the height of an amino acid within a stack indicates the relative frequency of that residue at that position. Below the logo are the sequences of the two heptad repeats found in SCR^14^. The sequence logo was created using WebLogo 3 (v.2.8.2). (B) The N-termini of SCR^+^ and SCR^14^ are predicted to have distinct secondary structures. SCR^14^ is predicted to encode a longer α-helical region than SCR^+^. (C) Helical wheel plot of the SCR^14^ N-terminus demonstrating the capacity for formation of an amphipathic helix. The circles of the wheel are labeled with letters *a* through *g*, designating the standard nomenclature of a heptad repeat of a coiled coil. The diagram illustrates hydrophobic amino acids in positions *a* and *d* (gray) and a general occupancy of positions *b*, *c*, and *f* (white) by uncharged, polar amino acids. Charged amino acids at *g* (blue) and *e* (red) are not present. Figure adapted from Nikolaev and Pervushin (2009). (D) Testing the capacity of SCR peptides to oligomerize. The sequence of the N-terminus of wild-type SCR is shown, highlighting the two putative heptad repeats spanning the octapeptide and LASCY motifs. The sequences are given for SCR peptides with substitutions in position *d* of one or both heptads, SCR^SL^ (SCR^+^) (i), SCR^LL^ (SCR^14^) (ii), SCR^LS^ (iii), and SCR^SS^ (iv). (E) Oligomerization is strongest with SCR^14^ peptides. SCR^14^ (SCR^LL^) peptides oligomerize strongly on addition of 1% (+) protein cross-linker, whereas SCR^+^ (SCR^SL^) peptides do not. (F) The ability of SCR peptides to oligomerize is dependent on the presence of two leucine residues. SCR peptides with point mutations in two putative heptad repeats were incubated with 1% (+) protein cross-linker. Removal of either or both leucine residues significantly reduced oligomerization. Proteins were detected on a Western blot using anti-FLAG antibody. Relative sizes of SCR^14^ oligomers, when run on 15% (E) and 12% (F) SDS-PAGE, are indicated in brackets.

An examination of the SCR^14^ N-terminus, encoding the octapeptide motif and LASCY motifs, revealed the presence of a putative leucine zipper motif based on three observations. First, we identified two putative heptad repeats in the SCR^14^ N-terminus; the same region in SCR^+^ encodes a single putative heptad repeat. A sequence logo compiled from leucine zipper motifs annotated in 27 Drosophila bZIP proteins identified by Fassler *et al.* (2002) revealed a general consensus sequence of E*_g_*L*_a_*E*_b_*A*_c_*L*_d_*R*_e_*Q*_f_* for a Drosophila heptad (Figure 2A) (Schneider and Stephens 1990; Fassler *et al.* 2002; Crooks *et al.* 2004). The two heptad repeats found in SCR^14^ both contain hydrophobic residues at positions *a* and *d*; however, charged residues are not present at residues *e* and *g*. When compared to SCR^14^, the sequence of the same region in SCR^+^ encodes a single putative heptad repeat spanning part of the octapeptide and the LASCY motifs (Figure 2A).

A second property of leucine zipper motifs is their amphipathic α-helical structure. The analysis of the SCR N-terminus using *ab initio* protein 2° structure prediction software identified α-helix in the region encoded by the octapeptide in both SCR^+^ and SCR^14^ using the Garnier-Robson method; however, this α-helix is slightly longer in SCR^14^ (data not shown; Protean v.4.0.3, DNA Star 1990–1999). The same software predicted α-helical region in the octapeptide of SCR^14^, but not SCR^+^, using the Chou-Fasman method. An alternative *ab initio* method for predicting the potential 2° protein structure is to search for the primary (1°) amino acid sequence of interest in the PDB of proteins with known 3D structure. This method predicted that the N-termini of SCR^+^ and SCR^14^ have distinct biochemical structures; where the SCR^+^ N-terminal encodes a short, α-helical region spanning part of the octapeptide and LASCY motif, the same region in SCR^14^ encodes a longer, α-helical region spanning both motifs entirely (Figure 2B; Supporting Information, Figure S1). A helical wheel plot of the SCR^14^ N-terminus predicts the formation of an amphipathic α-helix with a hydrophobic core formed by leucines at position *d* and hydrophobic residues at position *a*, and a hydrophilic surface formed primarily by noncharged, polar residues at positions *b*, *c*, and *f* (Figure 2C).

### *In vitro* analysis of SCR^14^

A third property of a leucine zipper motif is the capacity for oligomerization. To test the oligomerization capacity of SCR^+^ and SCR^14^ peptides, these peptides were expressed and purified from bacteria and incubated with and without protein cross-linker. First, it was noted that SCR^+^ and SCR^14^ ran at different relative molecular weights (M_r_). Second, the addition of protein cross-linker detected strong oligomerization of SCR^14^ peptides but not SCR^+^ peptides (Figure 2D). To identify the amino acids required for SCR^14^ oligomerization, modified SCR peptides were constructed with substitutions at the leucine residue present in each of the two putative heptad repeats of SCR^14^ (Figure 2C). Oligomerization of SCR^14^ peptides was dependent on the presence of two leucine residues, because removal of either or both leucines of the heptad repeats reduced oligomerization (Figure 2E). In addition, the M_r_ of modified SCR peptides was dependent on the number of serine residues present; there was an increase in M_r_ as leucines were substituted with serine residues.

### *In vivo* analysis of *Scr*^14^ antimorphy testing reciprocity

In sex comb formation, SCR^14^ inhibited SCR^+^ activity and the truncated SCR^13A^ protein inhibited SCR^14^ (Table 1). To determine if the inhibition of SCR^14^ by SCR^13A^ was reciprocal, truncated SCR^+^_121_TT and SCR^14^_121_TT peptides were expressed in *Scr*^+^ and *Scr*^14^ hemizygotes using the GAL4-UAS system. If the inhibition is reciprocal, then SCR^14^_121_TT is expected to inhibit endogenous SCR^+^ activity and SCR^+^_121_TT is expected to inhibit endogenous SCR^14^ activity, as observed with SCR^13A^. In the prothoracic segment, there was a significant effect of expressing SCR peptides in *Scr*^+^ hemizygotes [F_(2)_=8.4; *P* < 0.001] (Table 2). As expected, expression of SCR^14^_121_TT peptides significantly reduced the mean number of sex comb bristles from 6.4 to 5.5 (*P* < 0.001). A significant effect was also noted with expression of SCR peptides in the prothoracic segment of *Scr*^14^ hemizygotes [H_(2)_=5.7; *P* < 0.001]. As expected, expression of SCR^+^_121_TT peptides caused a significant decrease in the number of sex comb bristles from 3.0 to 1.3 (*P* < 0.001), and expression of SCR^14^ peptides also caused a significant decrease from 3.0 to 1.9 (*P* < 0.001). We also examined two other SCR-dependent phenotypes. In the proboscis, no effect was observed with expression of SCR_121_TT peptides in *Scr*^+^ hemizygotes [H_(2)_=5.7; *P* = 0.06]; however, there was a significant effect with expression of SCR peptides in *Scr*^14^ hemizygotes [F_(2)_=11.9; *P* < 0.001]. Expression of SCR^14^_121_TT peptides caused a significant increase in the number of rows of pseudotracheae from 3.1 to 3.6 (*P* = 0.01). In the salivary glands, there was a significant effect of expressing SCR peptides in *Scr*^+^ hemizygotes [F_(2)_=35.11; *P* < 0.001]. Expression of SCR^14^ peptides caused a significant decrease in the mean number of nuclei in the salivary gland from 113.3 to 91.9 (*P* < 0.001). A significant effect was also noted on expression of SCR peptides in the salivary glands of *Scr*^14^ hemizygotes [F_(2)_=19.6; *P* < 0.001]. Expression of SCR^14^ peptides caused a significant decrease in the mean number of nuclei from 100.7 to 88.8 (*P* < 0.001).

**Table 2:** Tissue-specific inhibition of endogenous *Scr* activity by ectopic expression of SCR^+^_121_TT or SCR^14^_121_TT polypeptides in flies hemizygous for *Scr*^+^ or *Scr*^14^ (±SEM)

UAS-X	Mean Sex Comb Bristles, No.		Mean Pseudotrachea, No.		Mean Cells in Salivary Gland, No.	
	*Scr^+^*	*Scr^14^*	*Scr^+^*	*Scr^14^*	*Scr^+^*	*Scr^14^*
None	6.4 ± 0.1*^a^*	3.0 ± 0.1*^a^*	5.3 ± 0.1*^a^*	3.1 ± 0.1*^a^*	113.3 ± 1.7*^a^*	100.7 ± 1.8*^a^*
*Scr^+^_121_TT*	6.0 ± 0.2*^a^*	1.3 ± 0.2*^b^*	5.5 ± 0.1*^a^*	3.0 ± 0.1*^a^*	114.5 ± 2.5*^a^*	105.0 ± 2.0*^a^*
*Scr^14^_121_TT*	5.5 ± 0.1*^b^*	1.9 ± 0.1*^c^*	5.6 ± 0.1*^a^*	3.6 ± 0.1*^b^*	91.9 ± 2.2*^b^*	88.8 ± 1.9*^b^*

Data in the same column with the same letters are not significantly different (*P* < 0.05).

### Heptad 2 is required for inhibition of SCR^14^

**Figure 3:**
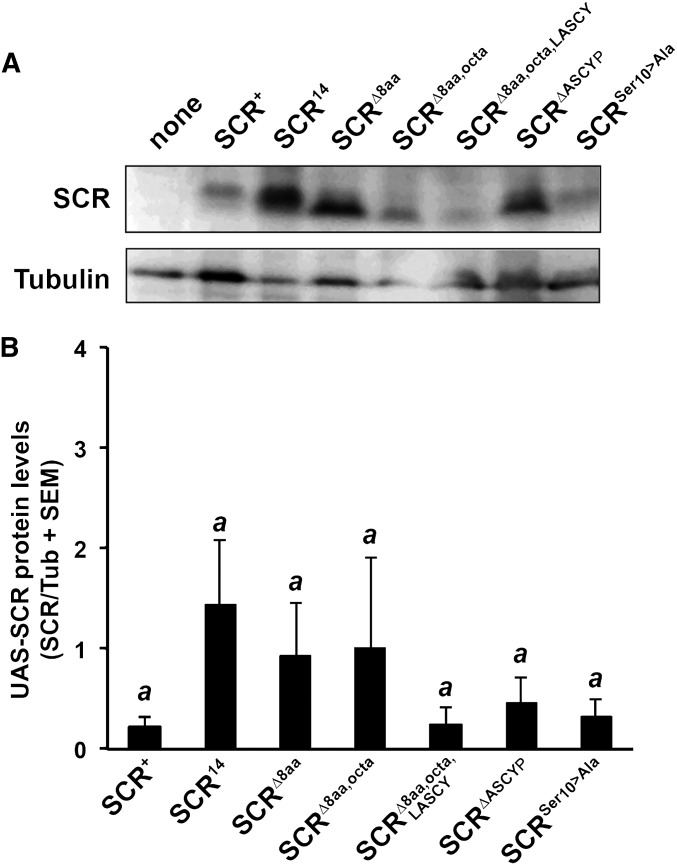
Expression of UAS-SCR^x^_121_TT constructs in Drosophila larvae. (A) Expression of tagged SCR peptides in third instar *Scr*^+^/*pb*^34^ larvae. (B) SCR peptide expression levels quantified from three independent Western blots. Protein levels are expressed as a ratio of SCR to tubulin, which was used as a loading control (SCR/tubulin).

**Figure 4:**
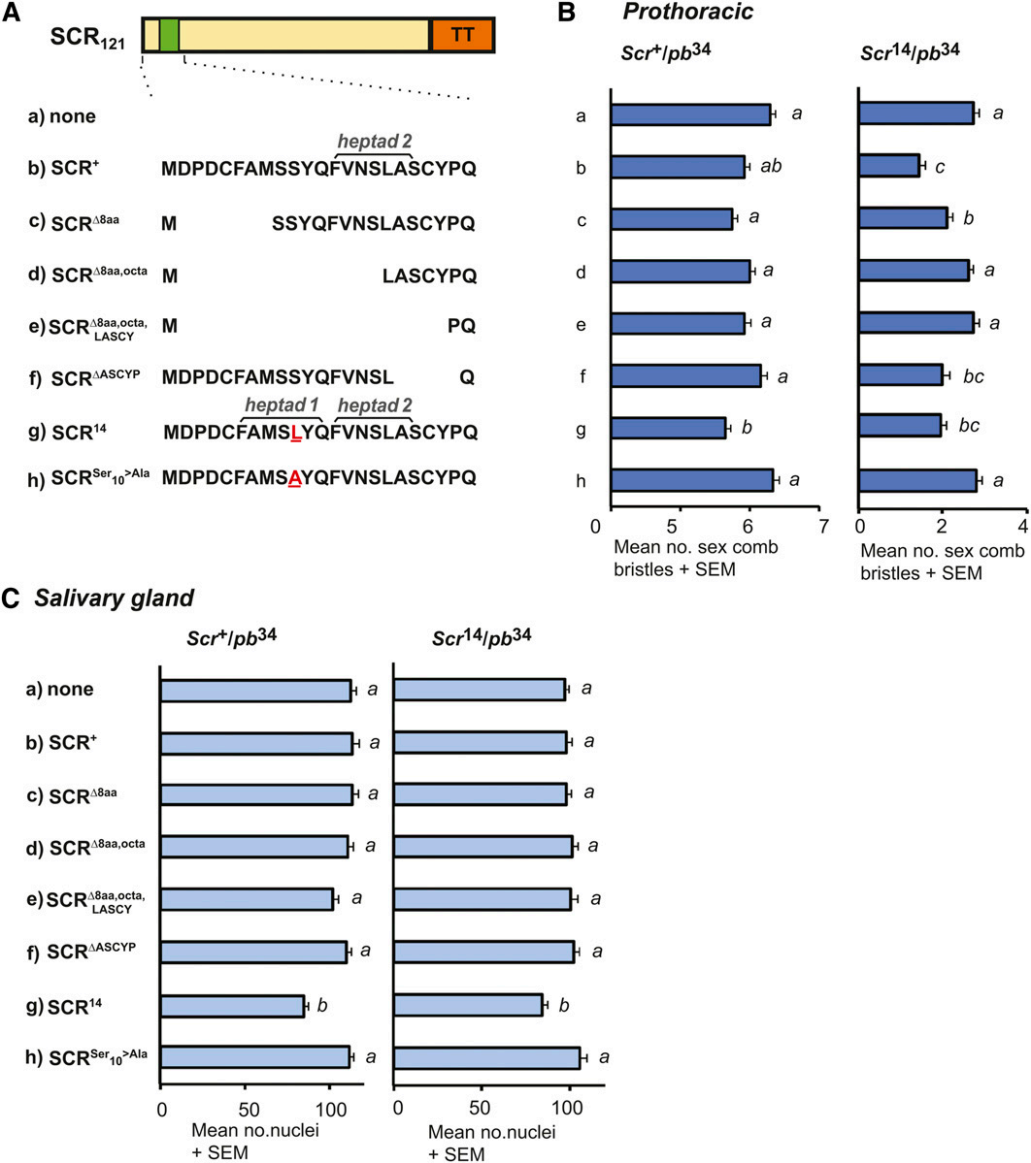
Inhibition of SCR activity requires heptad 2. Effect of expression of UAS-*Scr^x^*_121_TT constructs in two tissues: prothoracic segment and salivary glands. (A) UAS constructs made to identify the minimal sequence required for inhibiting SCR^14^ were deletions and point mutations (altered residues indicated in red) encompassing the octapeptide and LASCY motifs in the SCR N-terminus. (B) The effect of expression of UAS constructs on the development of the sex comb bristles in *Scr*^+^ and *Scr*^14^ hemizygotes. No UAS construct was control expression of GAL4 alone (a). (C) The effect of expression of UAS constructs on the development of salivary glands in *Scr*^+^ and *Scr*^14^ hemizygotes.

The observation that SCR^+^_121_TT peptides could inhibit SCR^14^ activity suggested that the second putative heptad, encoded by part of the octapeptide and the LASCY motif, is important for inhibition of SCR^14^ activity. To test this idea, altered SCR peptides: SCR^+^_121_TT, SCR^14^_121_TT, SCR^Δ8aa^_121_TT, SCR^Δ8aa, octa^_121_TT, SCR^Δ8aa, octa, LASCY^_121_TT, and SCR^ΔASCYP^_121_TT were expressed in *Scr*^+^ and *Scr*^14^ hemizygotes. In addition, we tested whether inhibition of SCR^+^ activity would occur after a Ser-to-Ala change (SCR^Ser10→Ala^_121_TT). The accumulation of altered SCR proteins was compared in three replicates and no significant differences were noted (Figure 3). The effect of expression of these SCR peptides was assayed in two tissues: the sex comb bristles and the salivary glands (Figure 4). For sex comb bristle formation, there was a statistically significant effect of expressing modified SCR peptides in *Scr*^+^ hemizygotes [F_(7)_=5.5; *P* < 0.001]. Inhibition of endogenous SCR^+^ activity occurred only with expression of SCR^14^_121_ from 6.4 to 5.5 (*P* < 0.001). All other modified SCR peptides did not produce a significant decrease in sex comb bristle number. In the reciprocal experiment, there was a statistically significant effect of expressing modified SCR peptides in *Scr*^14^ hemizygotes [F_(7)_=11.6; *P* < 0.001]. Inhibition of endogenous SCR^14^ by the expression of SCR^+^_121_TT was indicated by a significant decrease in the mean number of sex comb bristles from 2.7 to 1.4 (*P* < 0.001), by expression of SCR^14^_121_TT from 2.7 to 1.9 (*P* = 0.004), by expression of SCR^Δ8aa^_121_TT from 2.7 to 2.1 (*P* = 0.03), and by expression of SCR^ΔASCYP^_121_TT from 2.7 to 2.0 (*P* = 0.02). All other modified SCR peptides did not produce a significant decrease in sex comb bristle number. In the salivary glands, there was a significant effect on expression of SCR peptides in *Scr*^+^ hemizygotes [F_(7)_=8.5; *P* < 0.001]. Inhibition of SCR^+^ was observed with expression of SCR^14^_121_TT peptides, indicated by a significant decrease in the mean number of nuclei in a salivary gland from 111.7 to 84.3 (*P* < 0.001). A significant effect was noted on expression of SCR peptides in *Scr*^14^ hemizygotes [F_(7)_=4.5; *P* < 0.001]. SCR^14^ activity was affected by expression of SCR^14^_121_TT peptides indicated by a weak yet significant decrease in the mean number of nuclei in a salivary gland from 96.4 to 84.3 (*P* = 0.04).

## Discussion

### Molecular mechanism of *Scr*^14^ antimorphy

The term "antimorphic" was coined by Müller to describe mutant genes that have an effect that is antagonistic to that of the wild-type gene from which they were derived by mutation (Müller 1932). Here we test a possible mechanism to explain the antimorphic nature of an allele of *Scr*. An *ab initio* survey of the N-terminal region of SCR^14^ identified a putative leucine zipper motif spanning the octapeptide and LASCY motifs presenting a potential explanation for the observed antimorphy. To test this idea, we analyzed the 1° and predicted 2° structures of SCR^+^ and SCR^14^ N-termini and found that SCR^14^ is predicted to form an amphipathic α-helix. Next, *in vitro* cross-linking assays demonstrated that SCR^14^ peptides have a strong propensity to oligomerize that is lacking in SCR^+^ peptides and are dependent on the presence of leucine residues at position *d* of the heptads. The formation of higher-order oligomers by SCR^14^ peptides, rather than dimers, was not surprising given the absence of charged residues at positions *e* and *g* of each heptad, which are required for dimer specificity (Fassler *et al.* 2002).

*In vivo* experiments to characterize the properties of the *Scr*^14^ leucine zipper motif found that inhibition is reciprocal, because SCR^14^_121_TT peptides inhibit SCR^+^ activity and SCR^+^_121_TT peptides inhibit SCR^14^ activity. However, the pattern of reciprocal inhibition was observed only in sex comb formation and not in the other tissues tested, the salivary glands, and proboscis. In the salivary glands only inhibition of SCR^+^ by ectopic SCR^14^_121_TT expression is observed. This result may be a consequence of haploinsufficiency of the *Scr* locus for sex comb but not salivary gland development (Sivanantharajah and Percival-Smith 2009). In the proboscis, ectopic expression of SCR^14^_121_TT resulted in an increase in the number of rows of pseudotrachea. A recent study suggests that SCR has two distinct activities that determine the labial and prothoracic segmental identities; therefore, this positive interaction may reflect a distinct effect of SCR^14^ on SCR labial activity compared with SCR prothoracic activity (Percival-Smith *et al.* 2013).

Closer examination of the SCR N-terminus to pinpoint the minimal region required for inhibition of SCR^14^ found that inhibition requires the presence of heptad 2, which spans part of the octapeptide and the LASCY motif. There were two results that were not consistent with experimental expectations. First, we did not expect to see inhibition of SCR^14^ activity by SCR^ΔASCYP^_121_TT. However, this is likely because the five-amino-acid deletion in SCR^ΔASCYP^_121_TT fails to remove the second heptad motif; rather, the original heptad is replaced with another, which is also predicted to be α-helical in structure (Figure S2). Second, we did not see inhibition of SCR^14^ activity by SCR^Ser10→Ala^_121_TT, though this peptide encodes a hydrophobic residue at position *d* of heptad 1. Is it possible that the formation of asymmetric *d-d′* pairs between Leu and Ala residues affects dimerization stability in some way.

### *Scr*^14^ presents a novel mechanism of antimorphy

A classic mechanistic explanation of antimorphy is the dominant negative, where inactive mutant protein subunits retain the capacity to form complexes with wild-type proteins, resulting in inactive protein complexes (Herskowitz 1987). There are a number of examples of leucine zipper encoding proteins behaving in a dominant negative manner. For instance, truncated versions of the developmentally important Arabidopsis protein ERECTA, a leucine-repeat–rich receptor-like Ser/Thr kinase (LRR-RTK), encoding the LRR were able to inhibit normal ERECTA function in a dominant negative fashion (Shpak *et al.* 2003). The introduction of a point mutation into the LRR abolished this dominant negative effect, indicating that a functional leucine zipper motif is essential for the antimorphic activity of truncated ERECTA proteins. In another example, truncated forms of the Drosophila transcription factor Fos, encoding a leucine zipper motif, were able to inhibit wild-type Fos activity (Pierre *et al.* 2008). Truncated peptides also demonstrated the ability to form novel heterodimers with other leucine zipper encoding proteins. With both ERECTA and Fos, it is presumed that inhibition occurs by forming inactive heterodimeric complexes with normal, and sometimes novel, binding partners (Shpak *et al.* 2003; Pierre *et al.* 2008).

When compared to the above examples, *Scr*^14^ antimorphy is unique because the mode of antimorphy is not dominant negative. There is no evidence that wild-type SCR proteins have an active leucine zipper motif and form oligomers via the octapeptide motif *in vivo*. Rather, a missense mutation introduces a leucine zipper motif into the SCR N-terminus, resulting in an acquired capacity for inhibiting endogenous SCR activity. The ability of a single amino acid change in SCR^14^ to alter SCR activity is supported by the observation that deletions of either the octapeptide motif or the LASCY motif do not decrease SCR activity for inducing ectopic sex comb bristles on the T2 leg as drastically as the SCR^14^ substitution (Percival-Smith *et al.* 2013). We have been unsuccessful in a number of assays in detecting hetero-oligomerization between SCR^+^ and SCR^14^ peptides; therefore, there are two possible explanations for the inhibition noted in this study. First, SCR peptides may form interactions with endogenously produced SCR molecules to directly inhibit their activity. Second, SCR^14^ peptides may interact with a protein other than SCR to inhibit SCR activity.

### SCR N-terminus as a transcriptional activation domain

Our inability to capture wild-type homodimers of SCR *in vitro* indicates that the capacity to form strong, detectable dimers is a specific function of SCR^14^. A recent study found that SCR dimerizes on DNA through the HD (Papadopoulos *et al.* 2012). It is possible that in a wild-type cell, dimerization on DNA via the HD facilitates interaction between two wild-type SCR N-termini through the shared heptad 2. Such an interaction could be highly dependent on DNA binding for bringing the SCR N-termini into close proximity and for stability. Given the necessity for protein cross-linker in our *in vitro* experiments, it is likely that the SCR–SCR interactions observed in this study are transient and unstable. This lack of stability may have hampered our attempts to determine the structure of the SCR^+^ and SCR^14^ termini using NMR spectroscopy, which found these regions to be unstructured *in vitro* (Anthony Percival-Smith, unpublished observations). However, the SCR N-terminus may be structured *in vivo* given the observation that a large number of proteins are intrinsically disordered until bound to a target molecule (Wright and Dyson 1999; Dyson and Wright 2005; Cordier *et al.* 2006). The lack of intrinsic structure has a number of advantages: increased specificity, because binding of a protein to a target is dependent on an energy-induced transition change, and tight control over protein accumulation, because unstructured proteins are quickly degraded (Dyson and Wright 2005). Structural analyses of a number of different transcriptional activation domains (TAD) have revealed that many TADs are unstructured until bound to a target protein, whereupon they fold into amphipathic α-helices (Zor *et al.* 2002; Dyson and Wright 2005). A role of the SCR N-terminus in transcriptional activation has been proposed before. Deletion of a 17-amino-acid region, encompassing the octapeptide and LASCY motifs, decreased ectopic transcriptional activation of the SCR targets, *forkhead* and *CrebA*, in Drosophila embryos (Tour *et al.* 2005). Interestingly, a search for potential TADs found that the SCR^14^ N-terminus encodes a 9aa TAD that is commonly found in a number of yeast and animal transcription factors (Piskacek *et al.* 2007). This predicted 9aa TAD motif in SCR^14^ is MSLYQFVNS, which is the sequence of the octapeptide motif. Although a 9aa TAD motif was not found in the SCR^+^ N-terminus, it is possible that this region may encode a TAD with different biochemical properties.

### SCR N-terminus as an interaction interface

The *Scr*^14^ mutation maps to a universally conserved octapeptide region that has been maintained since the last common bilateran ancestor, whereas the adjacent LASCY motif is a more recently derived sequence and is present in only protostome SCR homologs. The LASCY motif may have been conserved to stabilize protein–protein interactions required for the octapeptide to function as a TAD. The fortuitous identification *Scr*^14^ has allowed us to capture a potential function of the SCR N-terminus in mediating protein–protein interactions. Evidence suggesting that the octapeptide and LASCY motifs function as a TAD implies that this region must at least make contacts with components of the transcriptional machinery. In an extension of this idea, the ability of SCR^14^ to inhibit endogenous SCR^+^ activity also presents a novel mechanism of SCR regulation by leucine zipper encoding proteins. This suggests that the region of the SCR N-terminus encoding the octapeptide and LASCY motifs may serve an evolutionarily conserved function as a general interaction interface. One class of proteins known to regulate HOX function and also encode leucine zippers are the polycomb group (Pc-G) and trithorax group (Trx-G) classes of chromatin-associated proteins (Stankunas *et al.* 1998; Crosby *et al.* 1999; Kal *et al.* 2000). It remains to be determined whether proteins of either group modulate SCR activity by making intermolecular interactions with the SCR N-terminus.

### The antimorphic–hypomorphic *Scr*^14^ allele

In this study, we reclassify *Scr*^14^ as an antimorphic–hypomorphic allele. From the time of initial sequencing of the *Scr*^14^ (Sivanantharajah and Percival-Smith 2009) to the time of this publication, there appears to be a significant change in phenotypic behavior of this allele. We initially reported wild-type behavior of *Scr*^14^ when hemizygous; however, we now observe that SCR^14^ has reduced protein activity demonstrating that it is hypomorphic. When we first observed wild-type phenotypes in the proboscis, prothoracic leg, and salivary gland, we sequenced the *Scr*^14^ allele (Figure 1F) to ensure that the genotype was correct and found that it was. Our conclusion is that the phenotypic change observed in this study relative to the study by Sivanantharajah and Percival-Smith (2009) is not the result of changes to the *Scr*^14^ locus. A possible explanation may be the loss of a phenotypic modifier of *Scr* activity, which we were not successful at recovering from the stock in 2013, from the original *Scr*^14^ stock used for phenotypic analysis in 2009. Despite the potential loss of this hypothetical modifier, the *Scr*^14^ allele used here in all our analyses retains its key antimorphic activity.

*Scr*^14^ is also a hypomorphic allele, meaning that that it has reduced wild-type function. This property allows determination of the tissues in which the octapeptide is normally required and determination of whether this motif is uniformly or differential required. If each region of SCR were uniformly required in all tissues, then the same allelic series would be expected for each tissue. In a previous characterization of highly conserved motifs of SCR, it was found that the DYTQL motif, YPWM motif, and CTD were differentially required for three tissues assayed (Sivanantharajah and Percival-Smith 2009). To determine how the octapeptide was required for *Scr* function, the position of *Scr*^14^ was determined in an allelic series with previously characterized hypomorphic alleles of *Scr* in the study by Sivanantharajah and Percival-Smith (2009). The rank order, from weakest to strongest *Scr* phenotype, in the salivary glands was *Scr^+/−^= Scr^7^= Scr^8^= Scr^5^=* ***Scr^6^****≥* ***Scr^15^*** **=** ***Scr^14^****=* ***Scr^3^***; in the proboscis, the order was *Scr^+/−^≥* *****Scr^6^******= Scr^7^> Scr^8^> **Scr^14^**> **Scr^15^**= Scr^5^= **Scr^3^***; and in the prothoracic segment, the order was *Scr^+/−^> Scr^7^> **Scr^14^**= **Scr^3^**= **Scr^6^**> **Scr^15^**= Scr^8^ = Scr^5^*. The rank order of alleles varied for each of the three tissues examined, indicating a differential requirement of each protein region in each tissue. Particularly important (indicated in bold) was the placement within the order of the alleles with changes in the octapeptide (*Scr^14^*), DYTQL motif (*Scr^15^*), YPWM motif (*Scr^3^*), and CTD (*Scr^6^*), demonstrating that these four conserved protein regions are differentially pleiotropic.

## Supplementary Material

Supporting Information

## References

[bib1] Berry M. Gehring W. J., 2000 Phosphorylation status of the SCR Homeodomain determines its functional activity: essential role for protein phosphatase 2A,B’. EMBO J. 19: 2946–2957 10856239 10.1093/emboj/19.12.2946 PMC203353

[bib2] Brand A. H. Perrimon N., 1993 Targeted gene expression as a means of altering cell fates and generating dominant phenotypes. Development 118: 401–415 8223268 10.1242/dev.118.2.401

[bib4] Burkhard P. Meier M. Lustig A., 2000 Design of a minimal protein oligomerization domain by a structural approach. Protein Sci. 9: 2294–2301 11206050 10.1110/ps.9.12.2294 PMC2144530

[bib5] Carroll S. B., 1995 Homeotic genes and the evolution of arthopods and chordates. Nature 376: 479–485 7637779 10.1038/376479a0

[bib6] Carroll S. B. Grenier J. K. Weatherbee S. D., 2005 From DNA to Diversity: Molecular Genetics and the Evolution of Animal Design, Blackwell Publishing, Oxford

[bib8] Cordier F. Hartmann B. Rogowski M. Affolter M. Grzesiek S., 2006 DNA recognition by the brinker repressor–an extreme case of coupling between binding and folding. J. Mol. Biol. 361: 659–672 16876822 10.1016/j.jmb.2006.06.045

[bib9] Crooks G. E. Hon G. Chandonia J. M. Brenner S. E., 2004 WebLogo: A sequence logo generator. Genome Res. 14: 1188–1190 15173120 10.1101/gr.849004 PMC419797

[bib10] Crosby M. A. Miller C. Alon T. Watson K. L. Verrijzer C. P., 1999 The trithorax group gene moira encodes a brahma-associated putative chromatin-remodeling factor in *Drosophila melanogaster*. Mol. Cell. Biol. 19: 1159–1170 9891050 10.1128/mcb.19.2.1159 PMC116045

[bib11] Curtis C. D. Brisson J. A. DeCamillis M. A. Shippy T. D. Brown S. J., 2001 Molecular characterization of Cephalothorax, the Tribolium ortholog of Sex combs reduced. Genesis 30: 12–20 11353513 10.1002/gene.1027

[bib12] Dyson H. J. Wright P. E., 2005 Intrinsically unstructured proteins and their functions. Nat. Rev. Mol. Cell Biol. 6: 197–208 15738986 10.1038/nrm1589

[bib13] Ellenberger T. Brandl C. Struhl K. Harrison S., 1992 The GCN4 basic region leucine zipper binds DNA as a dimer of uninterrupted helices: Crystal structure of the protein-DNA complex. Cell 71: 1223–1237 1473154 10.1016/s0092-8674(05)80070-4

[bib14] Fassler J. Landsman D. Acharya A. Moll J. R. Bonovich M., 2002 B-ZIP proteins encoded by the Drosophila genome: evaluation of potential dimerization partners. Genome Res. 12: 1190–1200 12176927 10.1101/gr.67902 PMC186634

[bib15] Galant R. Walsh C. M. Carroll S. B., 2002 Hox repression of a target gene: extradenticl-independent, additive action through multiple monomer binding sites. Development 129: 3115–3126 12070087 10.1242/dev.129.13.3115

[bib53] Henikoff S. Henikoff J. G., 1992 Amino acid substitution matrices from protein blocks. Proc Natl Acad Sci U S A. 89: 10915–10919 1438297 10.1073/pnas.89.22.10915 PMC50453

[bib16] Herskowitz I., 1987 Functional inactivation of genes by dominant negative mutations. Nature 329: 219–222 2442619 10.1038/329219a0

[bib17] Hittinger C. T. Stern D. L. Carroll S. B., 2005 Pleiotropic functions of a conserved insect-specific Hox peptide motif. Development 132: 5261–5270 16267091 10.1242/dev.02146

[bib18] Joshi R. Passner J. M. Rohs R. Jain R. Sosinsky A., 2007 Functional specificity of a Hox protein mediated by the recognition of minor groove structure. Cell 131: 530–543 17981120 10.1016/j.cell.2007.09.024 PMC2709780

[bib19] Kal A. J. Mahmoudi T. Zak N. B. Verrijzer C. P., 2000 The Drosophila brahma complex is an essential coactivator for the trithorax group protein zeste. Genes Dev. 14: 1058–1071 10809665 PMC316570

[bib20] Klymkowsky M. W. Maynell L. A. Polson A. G., 1987 Polar asymmetry in the organization of the cortical cytokeratin system of Xenopus laevis oocytes and embryos. Development 100: 543–557 2443336 10.1242/dev.100.3.543

[bib21] Krylov D. Mikhailenko I. Vinson C., 1994 A thermodynamic scale for leucine zipper stability and dimerization specificity: e and g interhelical interactions. EMBO J. 13: 2849–2861 8026470 10.1002/j.1460-2075.1994.tb06579.x PMC395166

[bib22] Landschulz W. H. Johnson P. F. McKnight S. L., 1988 The leucine zipper: a hypothetical structure common to a new class of DNA binding proteins. Science 240: 1759–1764 3289117 10.1126/science.3289117

[bib23] Lewis E. B., 1978 A gene complex controlling segmentation in Drosophila. Nature 276: 565–570 103000 10.1038/276565a0

[bib24] Lewis R. A. Wakimoto B. T. Denell R. E. Kaufman T. C., 1980 Genetic analysis of the Antennapedia gene complex (Ant-C) and adjacent chromosomal regions of Drosophila melanogaster. II. Polytene Chromosome segments 84A–84B1, 2. Genetics 95: 383–397 17249042 10.1093/genetics/95.2.383 PMC1214233

[bib25] Lindsley D. L. Zimm G. G., 1992 The Genome of Drosophila melanogaster. Academic Press, San Diego, California

[bib26] Merabet S. Litim-Mecheri I. Karlsson D. Dixit R. Saadaoui M., 2011 Insights into Hox protein function from a large scale combinatorial analysis of protein domains. PLoS Genet. 7: e1002302 22046139 10.1371/journal.pgen.1002302 PMC3203194

[bib27] McGinnis W. Krumlauf R., 1992 Homeobox genes and axial patterning. Cell 68: 283–302 1346368 10.1016/0092-8674(92)90471-n

[bib28] Müller, H. J., 1932 Further studies on the nature and causes of gene mutations. Sixth Int. Congr. Genet. 1: 213–255.

[bib29] Murre C. McCaw P. S. Vaessin H. Caudy M. Jan L. Y., 1989 Interactions between heterologous helix-loop-helix proteins generate complexes that bind specifically to a common DNA sequence. Cell 58: 537–544 2503252 10.1016/0092-8674(89)90434-0

[bib30] Nikolaev Y. Pervushin K., 2009 Rethinking Leucine Zipper–a ubiquitous signal transduction motif. Available from Nature Precedings: http://hdl.handle.net/10101/npre.2009.3271.1

[bib31] Panzer S. Weigel D. Beckendorf S. K., 1992 Organogenesis in *Drosophila melanogaster*: embryonic salivary gland determination is controlled by homeotic and dorsoventral patterning genes. Development 114: 49–57 1349523 10.1242/dev.114.1.49

[bib32] Papadopoulos D. K. Skouloudaki K. Adachi Y. Samakovlis C. Gehring W. J., 2012 Dimer formation via the homeodomain is required for function and specificity of Sex combs reduced in Drosophila. Dev. Biol. 367: 78–89 22564794 10.1016/j.ydbio.2012.04.021

[bib33] Percival-Smith A. Weber J. Gilfoyle E. Wilson P., 1997 Genetic characterization of the role of the two HOX proteins, Proboscipedia and Sex combs reduced, in determination of adult antennal, tarsal, maxillary palp and proboscis identities in *Drosophila melanogaster*. Development 124: 5049–5062 9362475 10.1242/dev.124.24.5049

[bib34] Percival-Smith A. Sivanantharajah L. Pelling J. J. Teft W. A., 2013 Developmental competence and the induction of ectopic proboscises in *Drosophila melanogaster*. Dev. Genes Evol. 223: 375–387 24121940 10.1007/s00427-013-0454-8

[bib35] Perera R. Owen K. E. Tellinghuisen T. L. Gorbalenya A. E. Kuhn R. J., 2001 Alphavirus nucleocapsid protein contains a putative coiled coil alpha-helix important for core assembly. J. Virol. 75: 1–10 11119567 10.1128/JVI.75.1.1-10.2001 PMC113891

[bib36] Pierre W. Morra R. Lucchesi J. Yedvobnick B., 2008 A male-specific effect of dominant-negative Fos. Dev. Dyn. 237: 3361–3372 18924113 10.1002/dvdy.21751

[bib52] Piskacek S. Gregor M. Nemethova M. Grabner M. Kovarik P. Piskacek M., 2007 Nine-amino-acid transactivation domain: establishment and prediction utilities. Genomics 89: 756–768 17467953 10.1016/j.ygeno.2007.02.003

[bib37] Prince F. Katsuyama T. Oshima Y. Plaza S. Resendez-Perez D., 2008 The YPWM motif link Antennapedia to the basal transcriptional machinery. Development 135: 1669–1679 18367556 10.1242/dev.018028

[bib38] Rubin G. M. Spradling A. C., 1982 Genetic transformation of Drosophila with transposable element vectors. Science 218: 348–353 6289436 10.1126/science.6289436

[bib39] Schneider T. D. Stephens R. M., 1990 Sequence logos: A new way to display consensus sequences. Nucleic Acids Res. 18: 6097–6100 2172928 10.1093/nar/18.20.6097 PMC332411

[bib40] Shpak E. D. Lakeman M. B. Torii K. U., 2003 Dominant-negative receptor uncovers redundancy in the Arabidopsis ERECTA Leucine-rich repeat receptor-like kinase signaling pathway that regulates organ shape. Plant Cell 15: 1095–1110 12724536 10.1105/tpc.010413 PMC153719

[bib41] Sivanantharajah L. Percival-Smith A., 2009 Analysis of the sequence and phenotype of Drosophila Sex combs reduced alleles reveals potential functions of conserved protein motifs of the Sex combs reduced protein. Genetics 182: 191–203 19293143 10.1534/genetics.109.100438 PMC2674816

[bib42] Stankunas K. Berger J. Ruse C. Sinclair D. A. Randazzo F., 1998 The Enhancer of Polycomb gene of Drosophila encodes a chromatin protein conserved in yeast and mammals. Development 125: 4055–4066 9735366 10.1242/dev.125.20.4055

[bib43] Struhl G., 1982 Genes controlling segmental specification in the Drosophila thorax. Proc. Natl. Acad. Sci. USA 79: 7380–7384 6961417 10.1073/pnas.79.23.7380 PMC347343

[bib44] Studier F. W. Moffatt B. A., 1986 Use of bacteriophage T7 RNA polymerase to direct selective high-level expression of cloned genes. J. Mol. Biol. 189: 113–130 3537305 10.1016/0022-2836(86)90385-2

[bib45] Studier F. W. Rosenberg A. H. Dunn J. J. Dubendorff J. W., 1990 Use of T7 RNA polymerase to direct expression of cloned genes. Methods Enzymol. 185: 60–89 2199796 10.1016/0076-6879(90)85008-c

[bib46] Tayyab I. Hallahan H. Percival-Smith A., 2004 Analysis of Drosophila proboscipedia mutant alleles. Genome 47: 600–609 15190377 10.1139/g03-133

[bib47] Tiefenbach J. Moll P. R. Nelson M. R. Hu C. Baev L., 2010 A live zebrafish-based screening system for human nuclear receptor ligand and cofactor discovery. PLoS ONE 5: 1–12 10.1371/journal.pone.0009797 PMC2842432 20339547

[bib48] Tour E. Hittinger C. T. McGinnis W., 2005 Evolutionary conserved domains required for activation and repression functions of the Drosophila Hox protein Ultrabithorax. Development 132: 5271–5281 16284118 10.1242/dev.02138

[bib49] Vinson C. Sigler P. McKnight S., 1989 A scissors-grip model for DNA recognition by a family of leucine zipper proteins. Science 246: 911–916 2683088 10.1126/science.2683088

[bib54] Wright P. E. Dyson H. J., 1999 Intrinsically unstructured proteins: re-assessing the protein structure-function paradigm. J. Mol. Biol. 293: 321–331 10550212 10.1006/jmbi.1999.3110

[bib50] Zhao J. J. Lazzarini R. A. Pick L., 1996 Functional dissection of the mouse *Hox-a5* gene. EMBO J. 15: 1313–1322 8635464 PMC450034

[bib51] Zor T. Mayr B. M. Dyson H. J. Montminy M. R. Wright P. E., 2002 Roles of phosphorylation and helix propensity in the binding of the KIX domain of CREB-binding protein by constitutive (c-Myb) and inducible (CREB) activators. J. Biol. Chem. 277: 42241–42248 12196545 10.1074/jbc.M207361200

