# Supplementary material for: Acquisition of a Leucine Zipper Motif as a Mechanism of Antimorphy for an Allele of the Drosophila Hox Gene Sex Combs Reduced
Source: G3 (Bethesda). 2014 Mar 12;4(5):829–38. doi: 10.1534/g3.114.010769 (PMC4025482; doi:10.1534/g3.114.010769)
Supplement: Supporting Information [file supp_4_5_829__index.html]

Acquisition of a Leucine Zipper Motif as a Mechanism of Antimorphy for an Allele of the Drosophila Hox Gene Sex Combs Reduced — Supporting Information 

# Acquisition of a Leucine Zipper Motif as a Mechanism of Antimorphy for an Allele of the *Drosophila Hox* Gene *Sex Combs Reduced*

## Supporting Information for Sivanantharajah and Percival-Smith, 2014

**Files in this Data Supplement:**

- Supporting Information - Figures S1-S2 (PDF, 262 KB)
- Figure S1 - *Ab initio* prediction of secondary protein structure. (PDF, 174 KB)
- Figure S2 - Predicted 2° structures of SCR+ and SCRΔASCYP. (PDF, 126 KB)
