# Supplementary material for: Acquisition of a Leucine Zipper Motif as a Mechanism of Antimorphy for an Allele of the Drosophila Hox Gene Sex Combs Reduced
Source: G3 (Bethesda). 2014 Mar 12;4(5):829–38. doi: 10.1534/g3.114.010769 (PMC4025482; doi:10.1534/g3.114.010769)
Supplement: Supporting Information [file supp_g3.114.010769_FigureS1.pdf]

A. SCR+

heptad 1 (g a b c d e f) *heptad 2* (g a b c d e f)

Query: FA**MSSYQFVNS**LASCY

Protein	Sequence	2° structure
Caps	FAMCSY	β-strand
PpcE	FGMSSYE	β-strand, coil
Nln	AMSSY	coil
Cox	AMSQYQ	coil
α-Amylase	MSSYDF	β-strand
Hmg1	MSSYAF	α-helix +coil
Qde2	MSSHQFV	α-helix, turn,β-strand
Aha1	SSYQF	coil
Bt_1439	SSYQF	coil
Alox12	SYQFLN	coil

B. SCR14

heptad 1 (g a b c d e f) *heptad 2* (g a b c d e f)

Query: FA**MSLYQFVNS**LASCY

Protein	Sequence	2° structure
LigAB	FAMSL	α-helix, coil
Vta1	FTMSLY	α-helix
Sden_2526	FAINLYQ	β-strand
Rad50	AMSLY	α-helix
Pbp1b	AMSIYQ	α-helix
Panc	ALSLYQ	α-helix, turn
Ylbp	MSLYQ	β-strand
Plc	MSLYQ	α-helix
Hda1	MSLYQ	α-helix, coil
Blf1	MSLYQY	β-strand
YaeB	MSLYEF	β-strand, turn, β-strand
Fen1	MSIYQF	α-helix
Annexin	SLYEEI	α-helix
TatD	SLFEFVN	α-helix

C. Shared

heptad 1 (g a b c d e f) *heptad 2* (g a b c d e f)

Query: FA**MSSYQFVNS**LASCY

Protein	Sequence	2° structure
DsbA	YQFVN	β-strand
Irf3	YEFVNS	β-strand
Aaci_2157	YPFVQSLA	β-strand, turn, α-helix
IL-1β	QFVSSL	β-strand
Top2	FVNSIA	β-strand, turn
GrE	FVNSLSS	α-helix
HsdS	YVNELAS	α-helix
vSGLT	VSSLAS	α-helix
Adc	VNSLAS	α-helix
Sat	VNSLGSC	coil
Klc2	NNSLASCY	α-helix
KdsC/Yrbl	SLATCY	α-helix
Arp8	SLATCY	β-strand, turn

Figure S1 *Ab initio* prediction of secondary protein structure. Matches for when SCR+ and SCR14 sequences were used as queries to search the NCBI pdb are indicated for the region encoded by SCR+ (A), SCR14 (B) and the region common to both proteins (C). The name of the protein, sequence of homology or identity, secondary (2) structure of each sequence in the solved protein, and the domain in which each sequence falls is indicated for each match. The proteins that were identified in our search were: Acetoacetate Decarboxylase (Adc; PDB ID: 3CMB_A), α -Amylase (1KXH_A), Interferon regulatory factor 3 (Irf3; 1T2K_A), Cytochrome C Oxidase (Cox; 1V54_A), Post-Proline Cleaving Enzyme (PpcE; 4HVT_A), Calcium- Dependent Activator Protein For Secretion (Caps; 1WI1_A), Co-chaperone protein (Aha1; 1USV_B), 3-deoxy-D-manno-octulosonate 8-phosphate phosphatase (KdsC; synonym: yrbl; 3I6B_A), Putative Susd-Like Carbohydrate Binding Protein (Bt_1439; 3SNX_A), Arachidonate 12-Lipoxygenase (Alox12; 3D3L_A), Actin-related Protein (Arp8; 4AM6_A), Neurolysin (Nln; 2O3E_A), High-mobility group protein B2 (Hmg1; 1CKT_A), Topoisomerase II (Top2; 1QZR_A), Kinesin Light Chain 2 (Klc2; 3ZFW_A), Type-1 restriction enzyme EcoKI specificity protein (HsdS; 2Y7C_B), Post-transcriptional gene silencing protein (Qde-2; 2XDY_A), *Interleukin-1* beta (IL-1β; 4G6J_A), Glycyl Radical Enzyme (GrE; 2Y8N_A), L-serine-o-acetyltransferase (Sat; 3VVM_A), N-Acetyltransferase (Ylbp; 2PR1_A), Disulfide oxidoreductase (DsbA; 3BD2_A), Burkholderia Lethal Factor 1 (Blf1; 3TUA_A), Putative tRNA methyltransferase (YaeB; 1XQB_A), Flap Endonuclease (Fen1; 3Q8M_A), Vacuolar protein sorting-associated protein (Vta1; 2LUH_A), Phosphatidylinositol-Specific Phospholipase C (Plc; 2PLC_A), Histone deacetylase HDA1 (Hda1; 3HGT_A), Sodium-glucose symporter (vSGLT; 3DH4_A), DNA repair protein (Rad50; 1US8_B), Annexin (1DM5_A), Tat-linked quality control protein (TatD; 3IPW_A), Transglycosylase penicillin-binding protein 1b (Pbp1b; 3VMA_A), Protocatechuate 4,5-dioxygenase (LigAB; 1BOU_A), Putative Metallopeptidase (Sden_2526; 3B2Y_A), Pantoate--Beta-Alanine Ligase (Panc; 2EJC_A), Xylose isomerase domain protein TIM barrel (Aaci_2157; 3U0H_A). The octapeptide motif is labeled in bold and the LASCY motif in italics. Above each query sequence, the residues constituting a heptad repeat are indicated.
