# Supplementary material for: Acquisition of a Leucine Zipper Motif as a Mechanism of Antimorphy for an Allele of the Drosophila Hox Gene Sex Combs Reduced
Source: G3 (Bethesda). 2014 Mar 12;4(5):829–38. doi: 10.1534/g3.114.010769 (PMC4025482; doi:10.1534/g3.114.010769)
Supplement: Supporting Information [file supp_g3.114.010769_FigureS2.pdf]

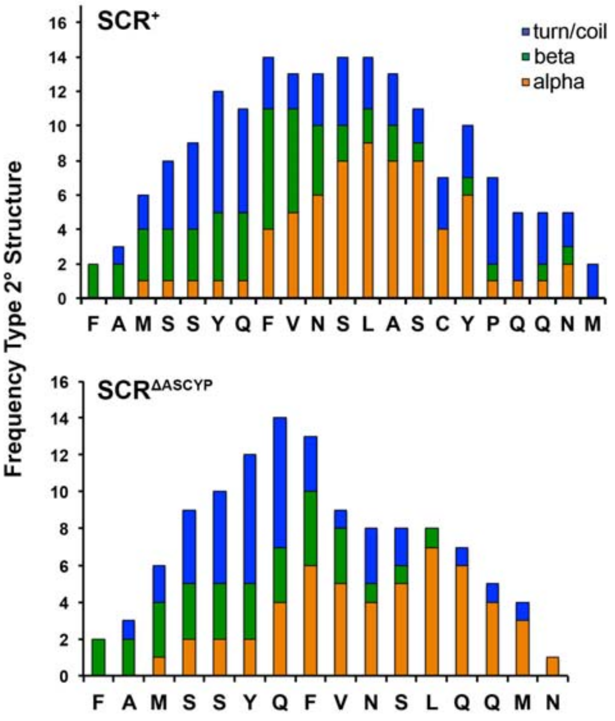

Figure S2 Predicted 2° structures of SCR+ and SCR$^{\Delta ASCYP}$**.** The five amino acid deletion in SCR$^{\Delta ASCYP}$ does not remove the heptad 2, rather it replaces it with another heptad that is also predicted to be alpha helical in structure.
